# Annales D’Oculistique

**Published:** 1896-01

**Authors:** 


ANNALES D'OCULISTIQUE, founded by Florent Cunier, and continued by Warlomont. Edited in Paris by Dr. D. E. Sulzer and Dr. E. Valude (French edition); in New York, by Dr. George T. Stevens (English edition). Fifty-eighth year. Volume CXIV, Nos. 1 to 5. The Transatlantic Publishing Co., New York, 63 Fifth Avenue; Paris, 198 Boulevard St. Germain; London, 26 Henrietta Street, Convent Garden. Five dollars per year.

The numbers for July, August, September, October, and November, 1895, of the English edition of this elegant periodical have been received at this office. The journal is issued simultaneously in Paris in French, and in New York in English, and contains the latest and best information on all subjects connected with the treatment of the eye. Each number has from sixty-four to eighty pages, and the whole year's issue makes two volumes, of about one thousand pages.

Some idea of its character may be had from the titles of some of the papers taken at random from the several numbers:

"Contribution to the Study of Œdematous Optic Neuritis of Inter-cranial Origin," by H. Parinaud; "Considerations upon the Application of Electrolysis to Twelve Cases of Detachment of the Retina," by Terson; "On Methodic Curetting of the Cornea in the Treatment of Pterygium," by Deschamps; "On the Curability of Sympathetic Uveitis," by Rognan; "Internal Sclerotomy," by de Wecker; "Ophthalmoscopic Examination with the Inverted Image in Highly Myopic Eyes," by Demicheri; "A Rare Endobulbar Tumor (Endothelial Sarcoma)," by Parisotti; "Hysterical Chromatism," by Pansier; "Hydraulic Curetting of the Cornea," by Santarnecchi; "The Possibility of Seeing One's Own Lens;" "Practical Utility of Phakoscopy in the Diagnosis of Fine Lens Opacities and in the Study of Development of Cataracts," by Darier; "Concerning a case of Suppurative Irido-choroiditis Terminating in Recovery," by Morax; "Pathogeny of Myopia," by Bitzos.

Many more of these titles might be quoted, but it is deemed that enough have here been given to give the reader a clear idea of the character of this periodical and its great value, not alone to the oculist, but to every cultivated physician.

